# Signet-ring cell carcinoma of the ampulla of Vater: a case diagnosed via repeated biopsies

**DOI:** 10.1007/s12328-020-01097-5

**Published:** 2020-01-24

**Authors:** Chisaki Ikeda, Naohiko Makino, Akiko Matsuda, Yasuharu Kakizaki, Tetsuya Ishizawa, Toshikazu Kobayashi, Shinpei Sugahara, Mayo Nishiduka, Michihiko Tsunoda, Junichiroh Haga, Rikiya Tsunoda, Yoshiyuki Ueno

**Affiliations:** 1 Department of Gastroenterology, Faculty of Medicine, Yamagata University, 2-2-2 Iidanishi, Yamagata, Yamagata 990-8595 Japan; 2 Department of Gastroenterology, Yonezawa City Hospital, 6-36 Aioichou, Yonezawa, Yamagata 992-8502 Japan; 3 Department of Surgery, Yonezawa City Hospital, 6-36 Aioichou, Yonezawa, Yamagata 992-8502 Japan; 4 Department of Pathology, Yonezawa City Hospital, 6-36 Aioichou, Yonezawa, Yamagata 992-8502 Japan

**Keywords:** Signet-ring cell carcinoma, Ampulla of Vater, Repeated biopsies

## Abstract

Signet-ring cell carcinoma of the ampulla of Vater is a rare tumor. A 74-year-old woman presented with epigastric pain and was diagnosed with cholangitis. Her liver enzyme levels were elevated. Computed tomography showed an enhanced area in the periampullary region and marked common bile duct dilatation. On endoscopic retrograde cholangiopancreatography (ERCP), the ampulla exhibited a normal appearance without ulcer or mass. Histological biopsy confirmed the absence of malignancy. During follow-up, the patient again presented with acute cholangitis multiple times and underwent ERCP each time. The ampulla had the appearance of a reddish and erosive mucosa. Although biopsy was repeated, histological examination did not show any malignancy. After a total of 13 biopsies, the patient was diagnosed with ampullary carcinoma of non-exposed protruded type following the third ERC-guided biopsy. Careful follow-up and frequent endoscopic biopsies are important in cases of papillary carcinoma of non-exposed protruded type with normal ampullary mucosa on initial endoscopy because this condition is challenging to diagnose with a single biopsy.

## Introduction

Signet-ring cell carcinoma (SRCC) mostly occurs in the stomach [1]; however, it is also observed in various organ systems including the gastrointestinal tract, hepato-pancreato-biliary, and urogenital systems [2]. Among them, SRCC originating from the ampulla of Vater is extremely rare [3]. Furthermore, papillary carcinoma of non-exposed protruded type can be difficult to diagnose histologically via endoscopic biopsy. Here we present the report of a patient who was diagnosed with SRCC of the ampulla of Vater via repeated biopsies.

## Case report

**Fig. 1:**
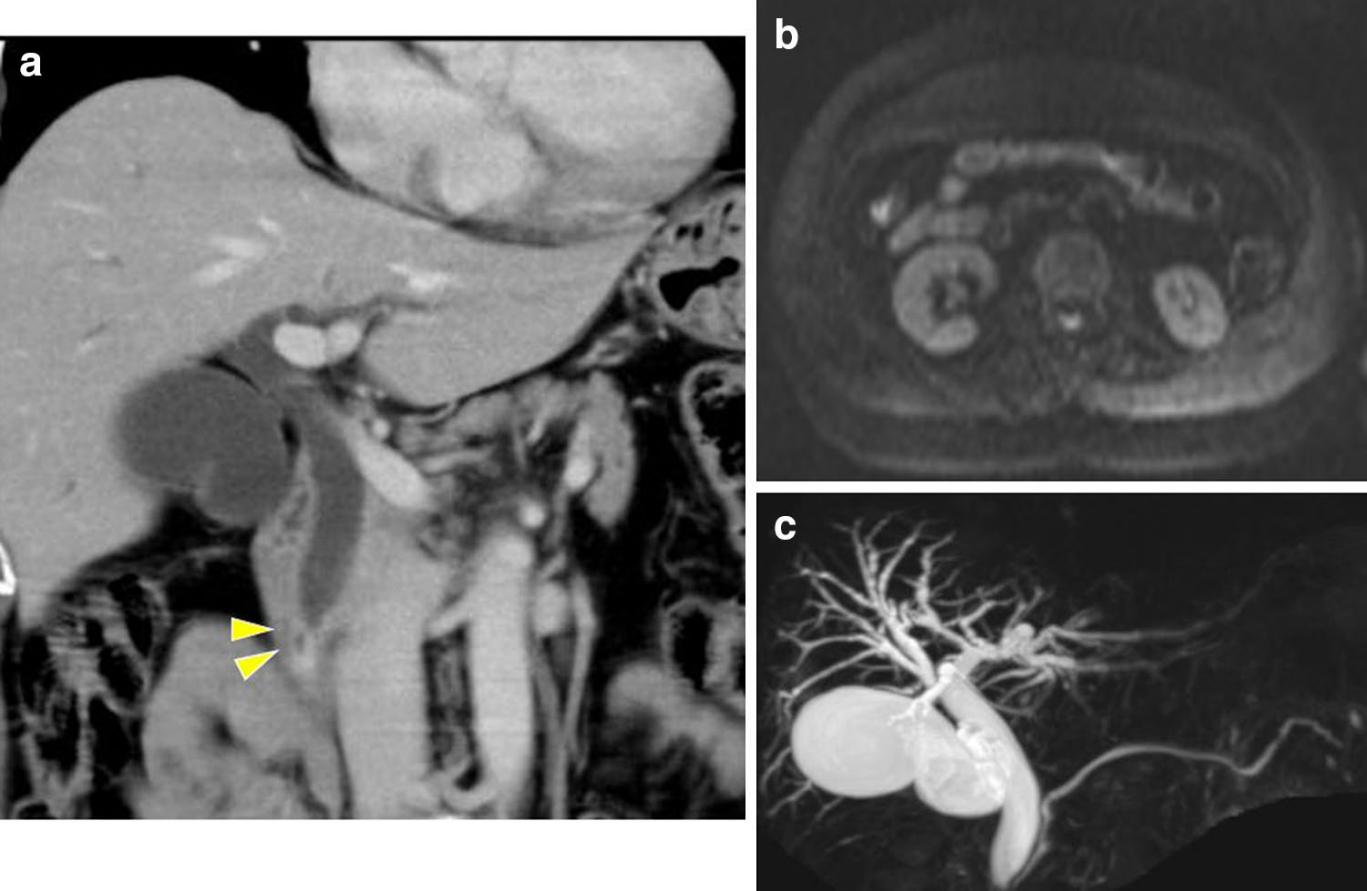
Imaging examinations. **a** Computed tomography shows a marked dilation of the common bile duct and an enhanced area in the periampullary lesion (yellow arrow head). **b** Magnetic resonance imaging (diffusion-weighted Imaging) showed no high-signal lesion in the ampulla of Vater. **c** Magnetic resonance cholangiopancreatography (MRCP) showed no significant main pancreatic duct dilation

A 74-year-old woman with abdominal pain was admitted to the hospital. Biochemical and hematological examinations revealed the following results, which indicated the presence of mild inflammation and liver dysfunction: alanine aminotransferase levels, 70 U/L; gamma-glutamyl transferase levels, 120 U/L; and C-reactive protein levels, 0.32 mg/dL. Carcinoembryonic antigen (CEA) and carbohydrate antigen (CA) 19-9 levels were within the normal range. Computed tomography (CT) showed dilation of the common bile duct and an enhanced area in the periampullary legion (Fig. 1a). Additionally, magnetic resonance imaging (MRI) showed no obvious mass lesion at this site (Fig. 1b). Magnetic resonance cholangiopancreatography (MRCP) showed no significant main pancreatic duct dilation (Fig. 1c).

**Fig. 2:**
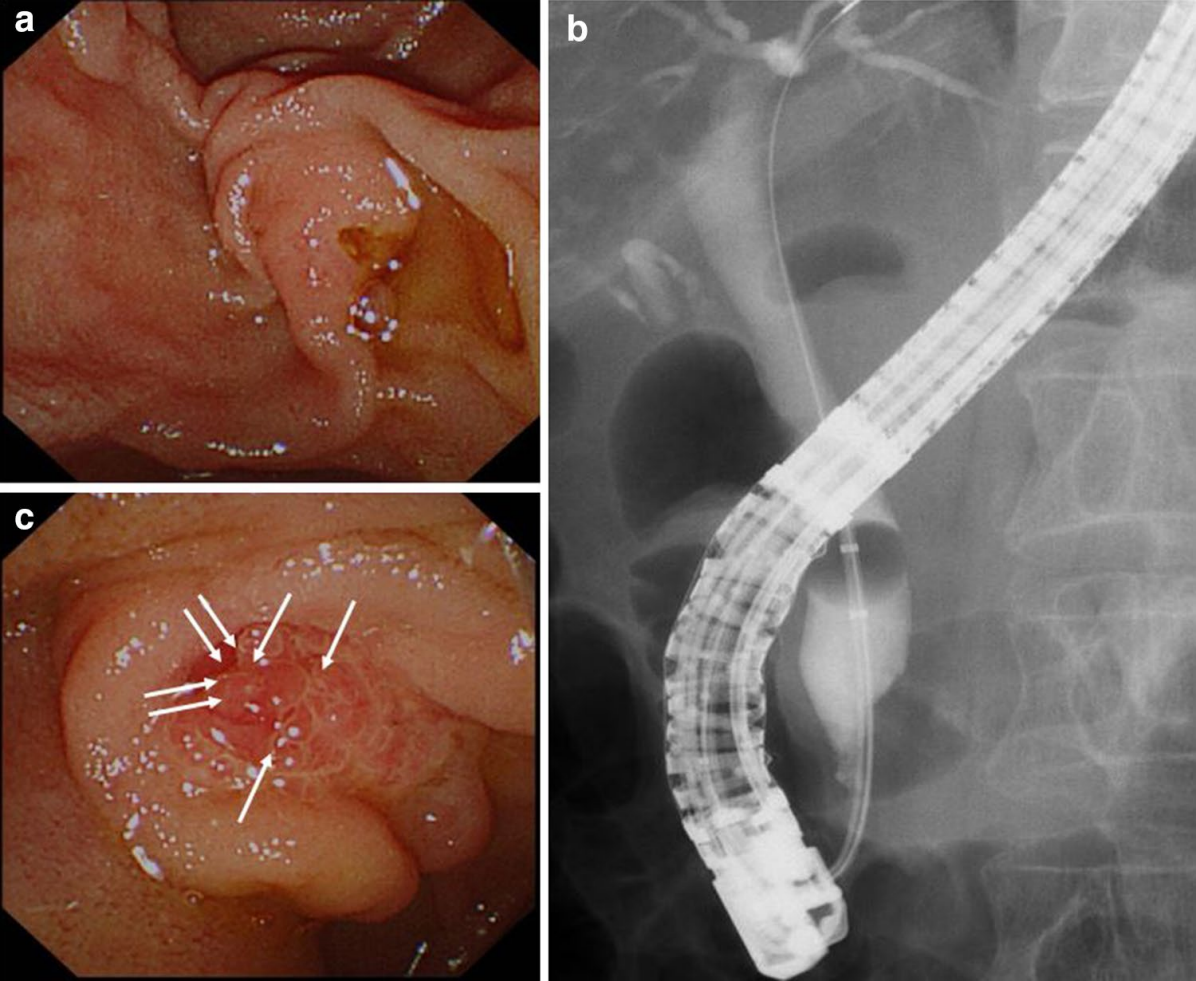
Endoscopic retrograde cholangiopancreatography (ERCP). **a** A normal ampulla of Vater was observed during the first ERCP. **b** Common bile duct dilatation was identified on cholangiogram; however, no obvious irregular stenosis was observed in the distal bile duct. **c** Ampulla of Vater exhibited redness and erosion several months later. Seven biopsies were performed during the second ERCP (white arrows)

**Fig. 3:**
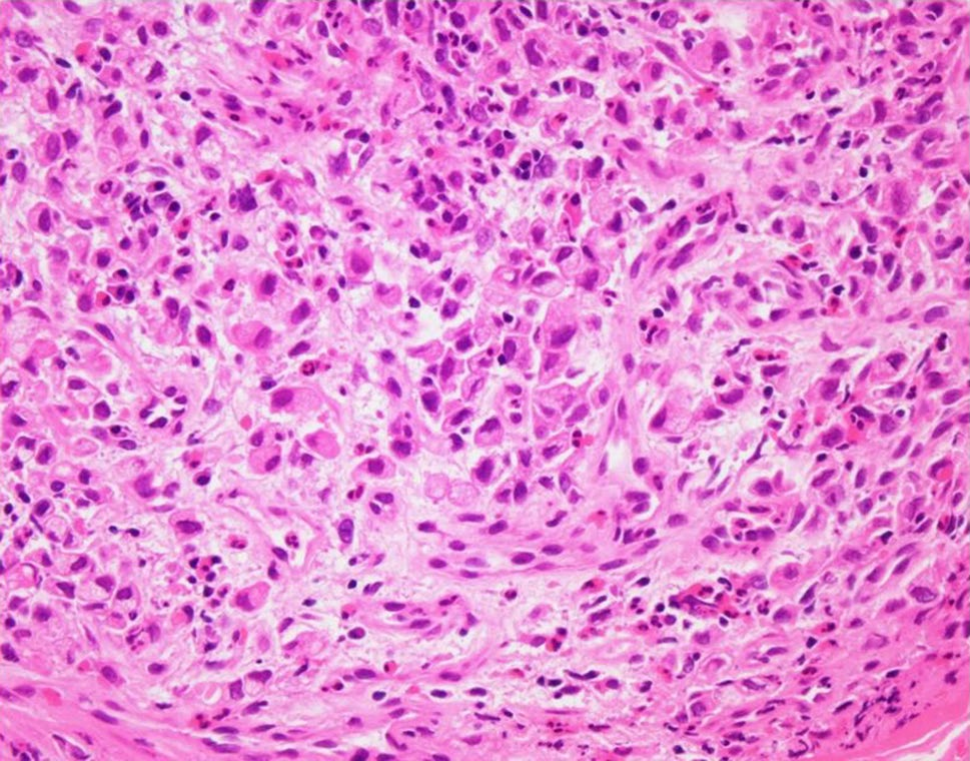
Histologic examination of the biopsied specimen. Adenocarcinoma with the characteristics of signet-ring cells (hematoxylin and eosin staining, × 40)

**Fig. 4:**
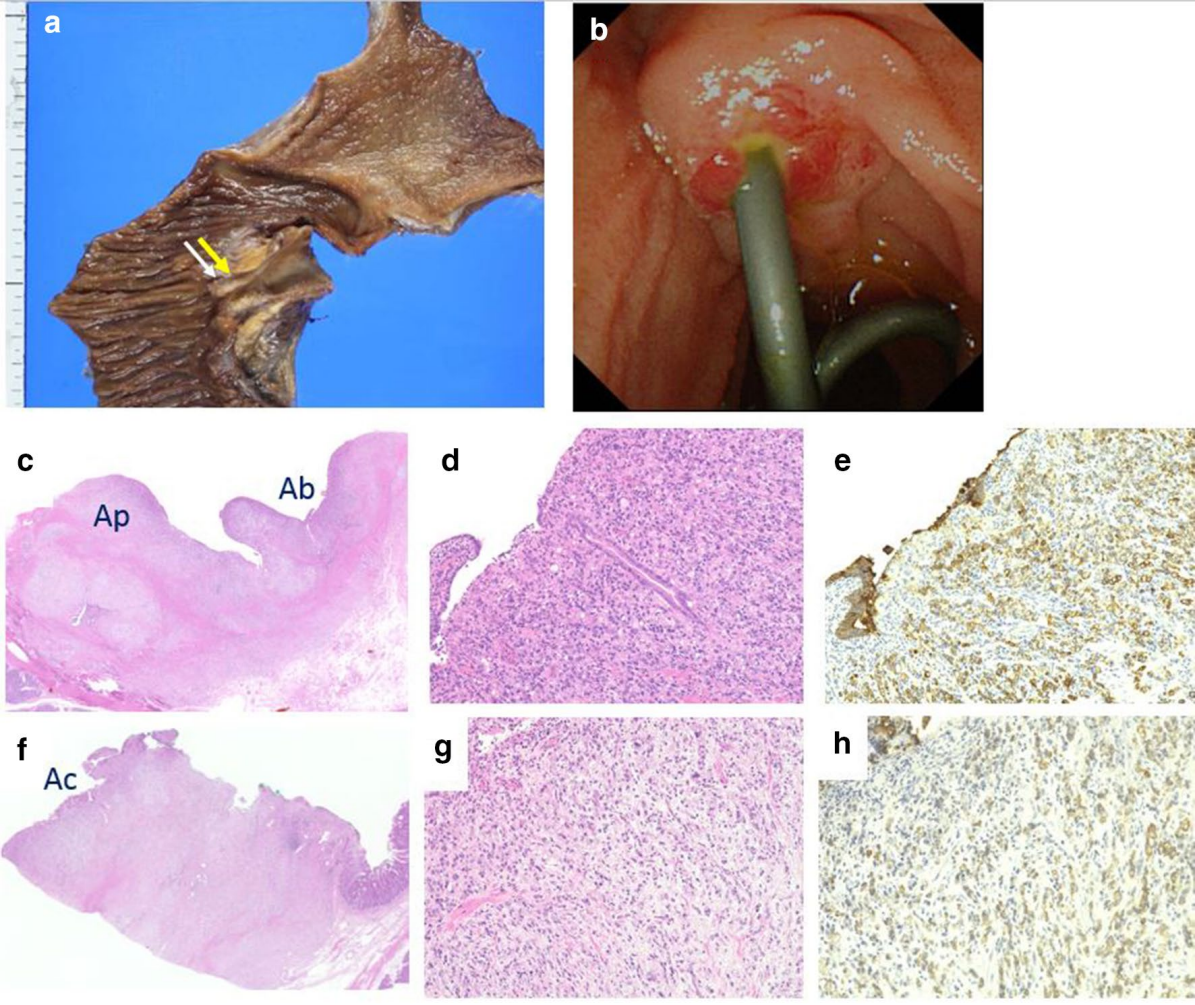
Resected specimen. **a** The tumor is located in the duodenal ampulla. The yellow arrow are sections of the ampullary bile duct region (Ab) and pancreatic duct region (Ap). The white arrow are sections of the ampullary common duct region (Ac). **b** Redness and erosion of the mucous membrane of the ampulla at the last ERCP. **c** The loupe image of yellow arrow’s section. **d**, **e** The microscopic image of Ab region (**d** hematoxylin and eosin staining, **e** cytokeratin AE1/AE3 staining, × 20). Signet-ring cell carcinoma was included in the epithelial components stained with AE1/AE3, and was mainly in Ab region. **f** The loupe image of white arrow’s section. **g**, **h** The microscopic image of Ac region (**g** hematoxylin and eosin staining, **h** cytokeratin AE1/AE3, × 20). Signet-ring cell carcinoma invasion and inflammatory cells

On endoscopic retrograde cholangiopancreatography (ERCP), the ampulla exhibited a normal appearance without ulcer or mass (Fig. 2a). However, bile duct cannulation was very difficult due to a long narrow distal segment, even though we tried three times using the pancreatic guidewire assisted cannulation [4]. Therefore, we performed precut sphincterotomy for deep selective bile duct cannulation. Cholangiography revealed common bile duct dilatation; however, no evident irregular stenosis was observed in the distal bile duct (Fig. 2b). Moreover, bile duct stones were not detected, and a small amount of biliary sludge was drained. Therefore we performed endoscopic sphincterotomy (EST), and four biopsies of the distal bile duct as well as cytology examination using a bile sample obtained using a nasal drainage tube for 3 days. However, no malignancy was observed. We suspected that cholangitis was caused by temporary biliary obstruction due to biliary sludge, papillary sphincter of Oddi dysfunction (SOD), or a malignant cancer. Therefore, the patient was cautiously monitored. Three months later, the patient was admitted to the hospital due to upper abdominal pain. She underwent ERCP as a treatment for acute cholangitis, and the results were similar to the previous cholangiography findings. However, the endoscopic image of the ampulla of Vater showed a reddish and erosive mucosa. Although we performed seven biopsies to detect a malignancy (Fig. 2c), no abnormal findings were observed. One month later, she again presented with the same symptoms. The ampulla of Vater remained reddish, and biopsies of the erosive mucosa (Fig. 4b) revealed an adenocarcinoma with the characteristics of signet-ring cells (Fig. 3). Preoperative imaging showed no evidence of metastatic disease, and the patient underwent a subtotal stomach-preserving pancreaticoduodenectomy. The resected specimen contained an 8 × 11-mm nodule in the ampulla of Vater (Fig. 4a). Microscopic examination of the ampullary tumor revealed a poorly differentiated adenocarcinoma with signet-ring cells that infiltrated the duodenal wall. Signet-ring cell carcinoma was included in the epithelial components stained with AE1/AE3. They were found in the ampullary bile duct (Ab) region, pancreatic duct (Ap) region, and common duct (Ac) region, and was mainly in the Ab region. There were also inflammatory cells in these areas (Fig. 4c–h). Invasion of the pancreatic parenchyma was not observed. The cancer stage was determined to be T2N0M0, Stage IB, according to the International Union Against Cancer TNM classification. The patient recovered well after surgery and did not receive adjuvant therapy. After approximately 6 months of follow-up, no evidence of recurrence was observed.

## Discussion

Tumors in the ampulla of Vater generally have a better prognosis and higher resection rates than other periampullary tumors [5]. Most ampullary tumors are well-differentiated adenocarcinomas, and poorly differentiated cancers, such as the SRCC of the ampulla of Vater, are uncommon. Gardner et al. first reported about SRCC as a variant of adenocarcinoma in 1990 [6]. Thus far, only 39 cases of this extremely rare malignancy have been reported and published in the medical literature [7, 8]. The pathogenesis, treatment, and outcome of this rare histologic subtype is not well known.

Although CT is performed for the diagnosis of SRCC, a dilated bile duct without a mass lesion in the ampulla of Vater is observed in some cases [9]. In ampullary tumor of the non-exposed protruded type, a sufficient sample for diagnosis is challenging to obtain because of a blind biopsy. Even skilled pathologists sometimes find it difficult to distinguish carcinomas from noninvasive lesions via forceps biopsy [10]. In the early stage, diagnosis made on the basis of endoscopic biopsy findings may not match the preoperative biopsy findings and the resected specimen of the ampullary tumor because the tissue might not have been obtained from the depth of the lesion where the cancer is present. Furthermore, endoscopic biopsy of the non-exposed protruded type has a false-negative rate of 50% [11].

Thus, depending on the type of ampullary cancer, endoscopic biopsy has a limited accuracy in the diagnosis of ampullary malignancy. Performing biopsy after EST and cannulation are useful methods for improving the accuracy rate [12]. Ogura et al. have reported that endoscopic ultrasound-fine needle aspiration may be safely and accurately performed for the lesions of the ampulla of Vater and that it should be preferred over EST as a diagnostic modality [13]. Lee et al. have reported about the need for re-evaluation using a side endoscope after the resolution of papillitis when endoscopic images and biopsy results are contrasting [14]. In addition, some reports have shown that the false-negative rate of the duodenoscopic appearance is only 14% [10]. Georgiotis et al. reported that the endoscopic appearance of the papilla was not normal in most patients; furthermore, even in cases with an abnormal appearance, all patients could be diagnosed via EST [15].

Advanced SRCC of the ampulla of Vater has a poor prognosis and is less chemosensitive than non-SRCC [16]. Lymph node infiltration is the most important prognostic factor of the SRCC of the ampulla [17]. Only about 30% of reported cases had no apparent recurrence during follow-up at 1 year [18]. SRCC distantly metastasizes to the liver [19], bone marrow [20], and leptomeninges [21]. There is no available adjuvant chemotherapy regimen for the SRCC of the ampulla [22]. In addition, the survival benefit of adjuvant therapy in patients without lymph node infiltration is unclear [23].

In our patient, we performed bile duct biopsies by collecting samples from 13 sites during ERCP as well as 7 biliary cytology examinations in about 5 months for cancer diagnosis. As several biopsies and cytology examinations showed no signs of malignancy and initial endoscopy showed a normal ampullary mucosa, we also suspected biliary-type SOD. Sphincterotomy for biliary-type SOD is reportedly effective in improving symptoms [24]. However, our patient experienced repeated abdominal pain and presented with acute cholangitis even after EST. Thus, the patient was cautiously followed up under suspicion of papillary carcinoma. We could not proceed to surgery because the pathological findings did not confirm the cancer. In addition, CT showed an enhanced area in the distal bile duct, therefore it was necessary to distinguish IgG4-SC. However, there was no suspicion of IgG4-related disease in other organs. We could not clearly exclude malignant tumors, so steroid testing was not performed and careful follow-up was performed.

We observed the morphological changes in the ampulla of Vater, and performed multiple biopsies. Finally, a diagnosis of SRCC was made. If it was difficult to diagnose periampullary lesion by multiple biopsies, diagnosis by EUS or IDUS had to be considered. The pathological findings of the surgical specimens showed that signet ring cell carcinoma was widely spread from the Ab region to the Ap and AC regions. Although distal bile duct showed mild wall thickening, no malignant findings. Therefore we diagnosed signet-ring cell carcinoma derived from the Ab region invaded the Ap and Ac region. Also it was thought that the tumor that invaded the Ac region during follow-up could be collected by performing biopsy many times. Moreover, in the Ac region, inflammatory cells that were thought to be due to effects after EST and biliary drainage tube placement were shown. Thus, we thought that the mucosal changes observed in the endoscopic findings were tumor infiltrating and inflammation.

In this case, surgery was performed approximately 5 months after the first visit; however, the lesion in the surgical specimen remained relatively small (11 mm). In addition, among the 39 reported cases of the SRCC of the ampulla, only 1 exhibited a normal appearing ampulla on ERCP and no mass on CT (Table.1). In a previous report, SRCC was detected via bone marrow biopsy; however, the patient did not present with jaundice and no abnormal findings were observed on ERCP. After 1 year, autopsy was performed, showing the presence of a 0.8-cm poorly differentiated adenocarcinoma of signet-ring cell type in the lamina propria of the ampulla of Vater [20]. In such a case, making a diagnosis via both imaging and histological examination would have been challenging because the condition was at an early stage.

**Table 1:** Reported cases of signet ring carcinoma of the ampulla of Vater

Author	References	Year	Age	Sex	Tumor size (mm)	CT findings	Endoscopic appearance of ampulla of Vater	T	N	M	Stage
Sekoguchi	[25]	1979	47	Male	20	NA	Invasion	3	0	0	IIA
Gardner	[6]	1990	69	Female	20	NA	Ulcerated	3	0	0	IIA
Tseng	[26]	2002	47	Male	20	NA	Ulcerated tumor	3	0	0	IIA
Hara	[17]	2002	68	Male	15	NA	Tumor	2	0	0	IB
Eriguchi	[27]	2003	83	Male	15	NA	Tumor	3	0	0	IIA
Nabeshima	[20]	2003	49	Male	8	No mass	Normal	3	X	1	IV
Fang	[28]	2004	53	Male	26	NA	Tumor	2	0	0	IB
Ramia	[23]	2004	67	Female	18	Mass	No tumor	2	0	0	IB
Li	[29]	2004	56	Female	15	No mass	Ulcerated	2	1	0	IIB
Valeri	[30]	2005	66	Male	NA	NA	NA	NA	NA	NA	NA
Purohit	[31]	2005	32	Female	NA	Deformed duodenum	Ulcerated	x	x	1	IV
Bloomston	[32]	2006	58	Female	10	NA	Tumor	2	0	0	IB
Akatsu	[18]	2007	43	Female	20	NA	Swollen	2	0	0	IB
Ishibashi	[33]	2009	59	Male	30	No mass	Irregularly shaped erosion	3	0	0	IIA
Gao	[9]	2009	38	Female	20	NA	Mass (endoscopic ultrasound)	3	0	0	IIA
Kim	[34]	2010	47	Male	30	NA	Tumor	x	1	x	III
Maekawa	[35]	2011	72	Male	20	Mass	Reddish swollen	3	0	0	IIA
Garcia	[36]	2011	74	Male	NA	NA	Tumor	3	0	x	IIA
Garcia	[36]	2011	73	Male	21	NA	Tumor	2	1	0	IIB
Paplomata	[21]	2011	45	Female	30	No mass	Tumor	4	1	0	III
Taş	[37]	2011	40	Male	NA	NA	Irregularly shaped erosion	X	1	1	IV
Gheza	[38]	2011	66	Male	5	NA	tumor	NA	NA	0	NA
Daoudi	[39]	2012	55	Male	NA	Mass	Irregularly shaped erosion	3	0	0	IIA
Lesquereux-Martinez	[40]	2012	78	Female	11	NA	NA	x	0	0	NA
Terada	[41]	2012	74	Female	NA	NA	Tumor	x	x	0	NA
Acharya	[42]	2013	78	Female	30	No mass	Tumor	3	0	0	IIA
Wen	[19]	2014	40	Female	30	Mass	NA	3	0	0	IIA
Wen	[19]	2014	64	Female	65	Mass	NA	4	x	0	III
Wen	[19]	2014	75	Female	35	Mass	NA	4	x	0	III
Wen	[19]	2014	62	Male	24	Mass	NA	x	1	0	IIB
Wen	[19]	2014	62	Male	30	Wall thickening	NA	x	1	0	IIB
Wen	[19]	2014	53	Male	12	Wall thickening	NA	3	0	0	IIA
Wen	[19]	2014	66	Female	15	Mass	NA	3	0	0	IIA
Wen	[19]	2014	68	Male	95	Mass	NA	4	x	0	III
Wakasugi	[22]	2015	59	Female	20	Mass	Swollen	3b	1	1	IV
Rahul	[43]	2016	53	Male	19	Mass	NA	4	1	0	III
Guus W.de Klein	[7]	2018	45	Female	12	NA	Swollen	2	0	0	IB
Ushida	[44]	2017	82	Female	22	Wall thickening	Swollen	3	0	0	IIA
Forneli	[8]	2019	49	Male	NA	NA	NA	NA	NA	NA	NA
Our case		2019	74	Female	11	No mass	Normal	2	0	0	IB

*NA* not available

No lymph node metastasis or distant metastasis was observed in this case; thus, postoperative chemotherapy was not administered. At present, no recurrence has been observed about 6 months after the operation. However, SRCC has a poor prognosis; thus, patients with SRCC should be cautiously followed up.

In summary, we presented the case of a patient with SRCC of the ampulla of Vater, which is an extremely rare condition. No mass was observed on CT, and a normal appearance was noted during the initial endoscopy. In cases with carcinoma of the ampulla, it may be difficult to rule out malignancy with just a single biopsy. In addition, patients with tumors in the ampulla of Vater may present with clinical symptoms that are extremely similar to those of SOD. Therefore, when SOD-like findings are observed multiple times after EST, repeated biopsy must be performed, which is important in ruling out ampullary carcinoma of non-exposed protruded type.

## References

[1] El-Zimaity HM, Itani K, Graham DY (1997). Early diagnosis of signet ring cell carcinoma of the stomach: role of the Genta stain. J Clin Pathol.

[2] Yokota T, Kunii Y, Teshima S (1998). Signet ring cell carcinoma of the stomach: a clinicopathological comparison with the other histological types. Tohoku J Exp Med.

[3] Howe JR, Klimstra DS, Moccia RD (1998). Factors predictive of survival in ampullary carcinoma. Ann Surg.

[4] Maeda S, Hayashi H, Hosokwa O (2003). Prospective randomized pilot trial of selective biliary cannulation using pancreatic guide-wire placement. Endoscopy.

[5] Morris-Stiff G, Alabraba E, Tan YM (2009). Assessment of survival advantage in ampullary carcinoma in relation to tumor biology and morphology. Eur J Surg Oncol.

[6] Gardner HA, Matthews J, Ciano PS (1990). A signet-ring cell carcinoma of the ampulla of Vater. Arch Pathol Lab Med.

[7] de Klein GW, van Baarlen J, Mekenkamp LJ (2018). Signet ring cell carcinoma of the ampulla of Vater: a rare histopathological variant. Case Rep Gastroenterol.

[8] Forneli A, Zanini N, De Biase D (2019). Signet ring cell carcinoma of the ampulla of Vater with focal neuroendocrine differentiation of the amphicrine type: report of a case with long-term survival. Int J Surg Pathol.

[9] Gao JM, Tang SS, Fu W (2009). Signet-ring cell carcinoma of ampulla of Vater: contrast-enhanced ultrasound findings. World J Gastroenterol.

[10] Deoliveira ML, Trivino T, de Jesus Lopes Filho G (2006). Carcinoma of the papilla of Vater: are endoscopic appearance and endoscopic biopsy discordant?. J Gastrointest Surg.

[11] Yamaguchi K, Enjyoji M, Kitamura K (1990). Endoscopic biopsy has limited accuracy in diagnosis of amupullary tumors. Gastrointest Endosc.

[12] Komorowski RA, Beggs BK, Greenan JE (1991). Assessment of ampulla of Vater pathology. An endoscopic approach. Am J Surg Pathol.

[13] Ogura T, Hara K, Hijioka S (2012). Can endoscopic ultrasound-guided fine needle aspiration offer clinical benefit for tumors of the ampulla of Vater? An initial study. Endosc Ultrasound.

[14] Lee HS, Jang JS, Lee S (2015). Diagnostic accuracy of the initial endoscopy for ampullary tumors. Clin Endosc..

[15] Skordilis P, Mouzas IA, Dimoulios PD (2002). Is endosonography an effective method for detection and local staging of the amupullary carcinoma? A prospective study. BMC Surg.

[16] Pernot S, Voron T, Perkins G (2015). Signet-ring cell carcinoma of the stomach: impact on prognosis and specific therapeutic challenge. World J Gastroenterol.

[17] Hara T, Kawashima H, Ishigooka M (2002). Signet-ring-cell carcinoma of the ampulla of Vater: a case report. Hepatogastroenterology.

[18] Akatsu T, Aiura K, Takahashi S (2007). Signet-ring cell carcinoma of the ampulla of Vater: report of a case. Surg Today.

[19] Wen X, Wu W, Wang B (2014). Signet ring cell carcinoma of the ampulla of Vater: immunophenotype and differentiation. Oncol Lett.

[20] Nabeshima S, Kishihara Y, Nabeshima A (2003). Poorly differentiated adenocarcinoma with signet-ring cells of the Vater’s ampulla, without jaundice but with disseminated carcinomatosis. Fukuoka Igaku Zasshi.

[21] Paplomata E, Wilfong L (2011). Signet ring cell carcinoma of the ampulla of Vater with leptomeningeal metastases: a case report. J Clin Oncol.

[22] Wakasugi M, Tanemura M, Furukawa K (2015). Signet ring cell carcinoma of the ampulla of Vater: report of a case and a review of the literature. Int J Surg Case Rep.

[23] Ramia JM, Mansilla A, Villar J (2004). Signet-ring cell carcinoma of the Vater’s ampulla. JOP.

[24] Craig AG, Toouli J (2001). Sphincterotomy for biliary sphincter of Oddi dysfunction. Cochrane Database Syst Rev.

[25] Sekoguchi T, Mizumoto R (1979). Clinicopathological study of papilla of Vater. Geka Chiryo.

[26] Tseng LJ, Jao YT, Mo LR (2002). Signet ring cell carcinoma of major papilla. Gastrointest Endosc.

[27] Eriguchi N, Aoyagi S, Jimi A (2003). Signet-ring cell carcinoma of the ampulla of Vater: report of a case. Surg Today.

[28] Fang CL, Chu JS, Hsieh MC (2004). Signet-ring cell carcinoma of the ampulla of Vater. J Formos Med Assoc.

[29] Li L, Chen QH, Sullivan JD (2004). Signet-ring cell carcinoma of the ampulla of Vater. Ann Clin Lab Sci.

[30] Valeri S, Caricato M, Ripetti V (2005). Signet-ring cell carcinoma of the Vater’s ampulla: report of a clinical case. Suppl Tumori.

[31] Purohit RC, Kant K, Bhargava N (2005). Signet ring cell carcinoma of ampulla of Vater in a young adult. Indian J Gastroenterol.

[32] Bloomston M, Walker M, Frankel WL (2006). Radical resection in signet ring carcinoma of the ampulla of Vater: report of an 11-year survivor. Am Surg.

[33] Ishibashi Y, Ito Y, Omori K (2009). Signet ring cell carcinoma of the ampulla of Vater. A case report. JOP.

[34] Kim DI, Park SW, Lee GS (2010). A case of signet-ring cell carcinoma of the ampulla of Vater. Korean J Gastroint Endosc.

[35] Maekawa H, Sakurada M, Orita H (2011). Signet-ring cell carcinoma co-existing with adenocarcinoma of the ampulla of vater. A case report. JOP.

[36] García AB, Arranz EM, Sanz RR (2011). Signet ring cell carcinoma of the ampulla of Vater. Gastroenterol Hepatol.

[37] Taş A, Ozer E, Köklü S (2011). Signet ring cell carcinoma of the ampulla of Vater: rare cause of acute pancreatitis. Scand J Gastroenterol.

[38] Gheza F, Ceryi E, Pulcini G (2011). Signet ring cell carcinoma of the ampulla of Vater: demonstration of pancreatobiliary origin. Pancreas.

[39] Daoudi K, El Haoudi K, Bouyahia N (2012). Signet ring cell carcinoma of the Vater’s ampulla: a very rare malignancy. Case Rep Oncol Med.

[40] Lesquereux-Martínez L, Fernández-Pérez A, Bustamante-Montalvo M (2012). Signet ring cell adenocarcinoma of the ampulla of Vater: a rare pathology. Rev Esp Enferm Dig.

[41] Terada T (2012). Primary signet-ring cell carcinoma of the ampulla of Vater: a case report with an immunohistochemical study. Appl Immunohistochem Mol Morphol.

[42] Acharya MN, Panagiotopoulos N, Cohen P (2013). Poorly-differentiated signet-ring cell carcinoma of the ampulla of Vater: report of a rare malignancy. JOP.

[43] Rahul D, Joshua W, Andrei C (2016). Signet ring cell carcinoma of the ampulla of Vater with early development of bone metastasis: case report and review of the rare malignancy. J Gastrointest Cancer.

[44] Ushida Y, Hiramatsu K, Saeki S (2017). Poorly differentiated adenocarcinoma with signet-ring cells in duodenal papilla: a case report. Surg Case Rep.

